# Supplementary figures and images for: Perception of four intellectual and developmental disabilities based on search engine and news portrayal
Source: PLoS One. 2025 Feb 10;20(2):e0316928. doi: 10.1371/journal.pone.0316928 (PMC11809880; doi:10.1371/journal.pone.0316928)

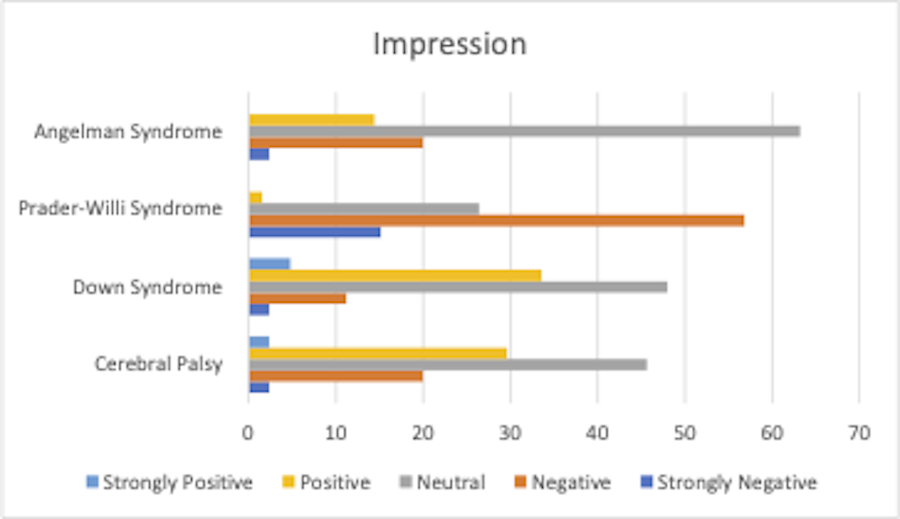

Supplement: S1 Fig — Frequencies of each response to the impression question for each condition. (TIF) [file pone.0316928.s001.tif]

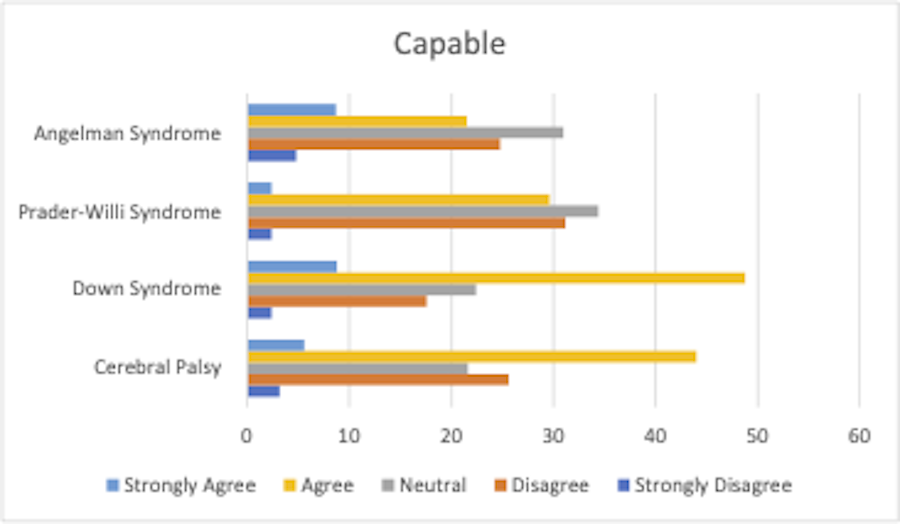

Supplement: S2 Fig — Frequencies of each response to the capable question for each condition. (TIF) [file pone.0316928.s002.tif]

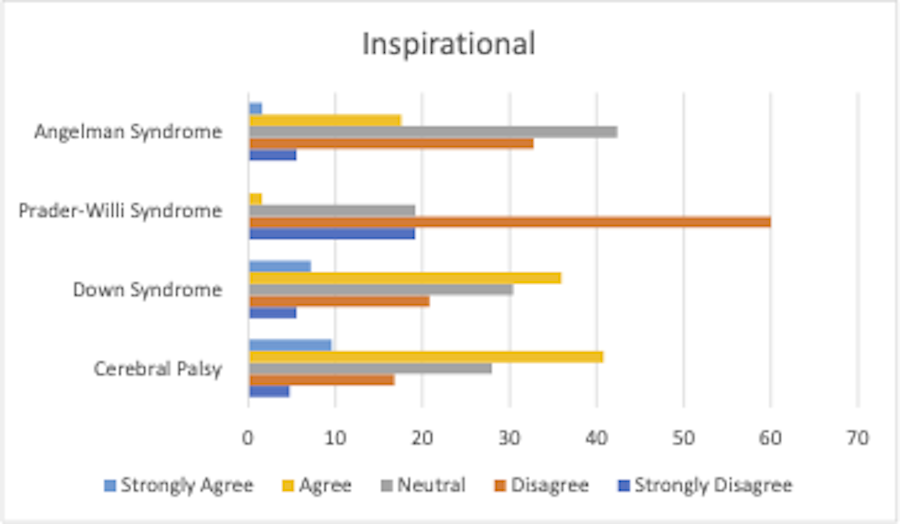

Supplement: S3 Fig — Frequencies of each response to the inspirational question for each condition. (TIF) [file pone.0316928.s003.tif]

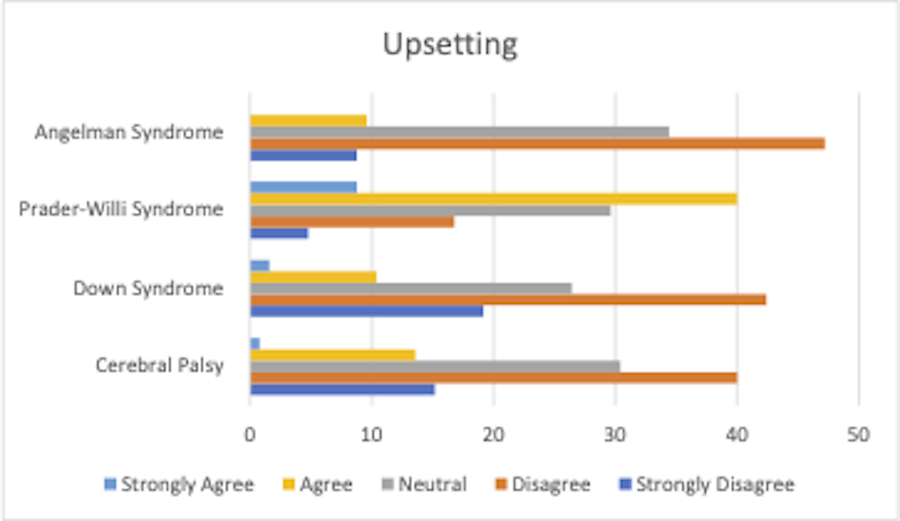

Supplement: S4 Fig — Frequencies of each response to the upsetting question for each condition. (TIF) [file pone.0316928.s004.tif]

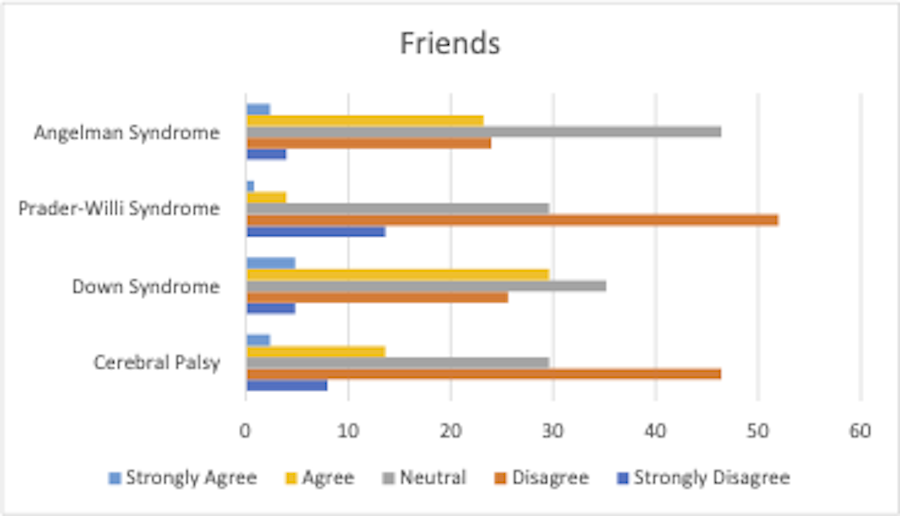

Supplement: S5 Fig — Frequencies of each response to the friends question for each condition. (TIF) [file pone.0316928.s005.tif]

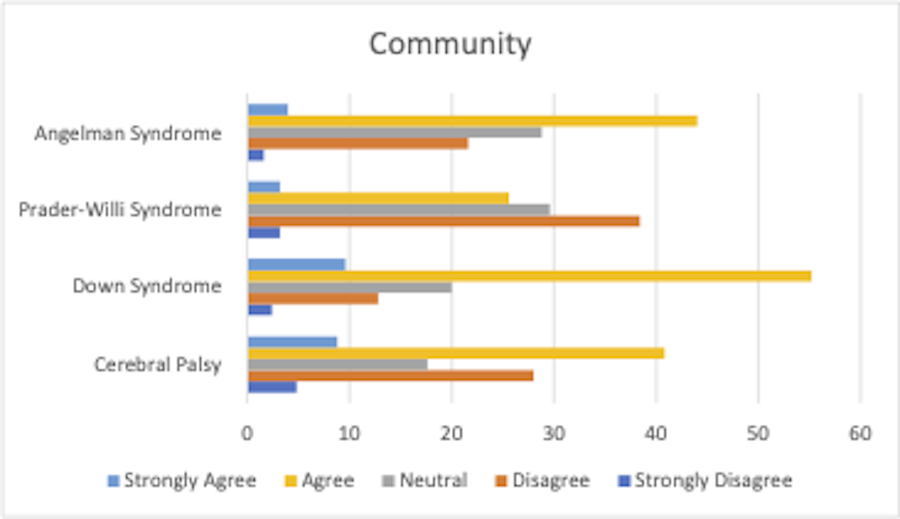

Supplement: S6 Fig — Frequencies of each response to the community question for each condition. (TIF) [file pone.0316928.s006.tif]

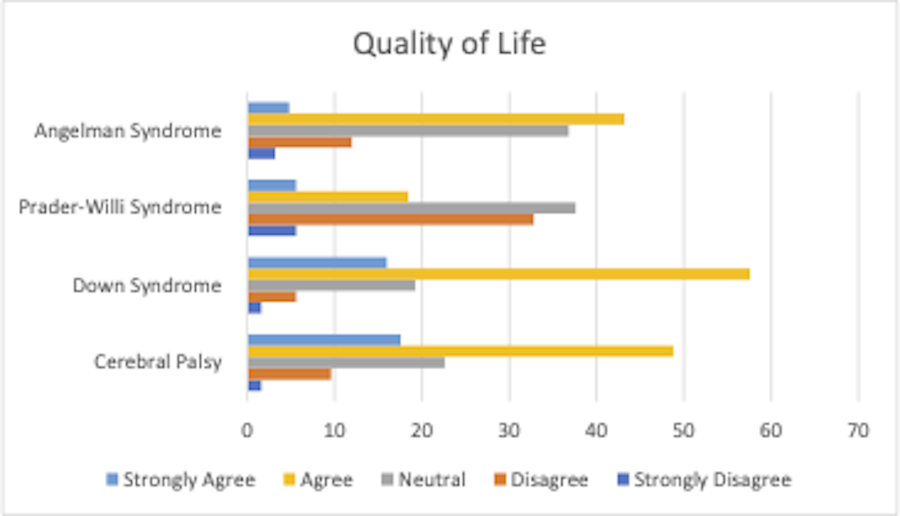

Supplement: S7 Fig — Frequencies of each response to the quality of life question for each condition. (TIF) [file pone.0316928.s007.tif]

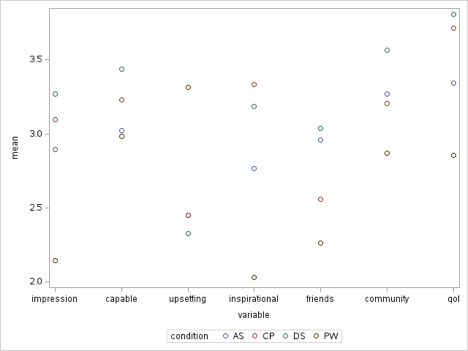

Supplement: S8 Fig — Plot of mean response to each question variable, grouped by condition. (TIF) [file pone.0316928.s008.tif]
