# Supplementary material for: Perception of four intellectual and developmental disabilities based on search engine and news portrayal
Source: PLoS One. 2025 Feb 10;20(2):e0316928. doi: 10.1371/journal.pone.0316928 (PMC11809880; doi:10.1371/journal.pone.0316928)
Supplement: S1 File — The full survey transcript, with all questions asked of participants. (DOCX) [file pone.0316928.s009.docx]

**Survey Transcript**

You are being asked to participate in a research study. We are doing this study to understand how different disabilities are portrayed and perceived in media. If you agree, we will ask you to take a survey containing images of Google search results and news articles, and react to them. The survey has 16 questions and should take 5 minutes to complete. We will not record your name or any information that shows your identity. Only the research team will see your answers and your information will remain private. If you have any questions, please contact Lillian Droscha by email ldroscha@bu.edu. By clicking "Agree" below, you are agreeing for the use and sharing of your survey answers.

Please confirm you are over age 18.

- Yes, I am over 18.
- No, I am not over 18.

Do you consent to participation in the survey?

- Yes
- No

Thank you for consenting to take our survey. We are interested in learning more about the portrayal of certain disabilities by search engines and news articles. This survey should take about 5 minutes to complete. We will ask you to examine top Google search results, images, and news articles for a variety of conditions, and react to them. Please answer the questions following the images based ONLY on the images provided, not any prior knowledge you may/may not have pertaining to a particular condition. Thank you again for taking the time to complete our survey!

Please carefully review the news headlines below related to Cerebral Palsy.

Please review the following search results related to Cerebral Palsy.

Please review the following images related to Cerebral Palsy.

Based ONLY on the search results and images presented above, my impression of Cerebral Palsy is:

- Strongly negative
- Negative
- Neutral
- Positive
- Strongly positive

Based ONLY on the search results and images presented above, select the level to which you agree or disagree with the following statements.

1. Someone with Cerebral Palsy is likely to be capable.
2. I find these images/search results inspirational.
3. I find these images/search results upsetting.

*Strongly agree, agree, neutral, disagree, strongly disagree

Based ONLY on the images presented above, select the level to which you agree or disagree with the following statements.

1. Someone with Cerebral Palsy likely has an easy time making friends.
2. I could envision someone with Cerebral Palsy engaging in multiple activities within my community.
3. It is possible for someone with Cerebral Palsy to have a high quality of life.

*Strongly agree, agree, neutral, disagree, strongly disagree

Please carefully review the news headlines below related to Down Syndrome.

Please review the following search results related to Down Syndrome.

Please review the following images related to Down Syndrome.

Based ONLY on the search results and images presented above, my impression of Down Syndrome is:

- Strongly negative
- Negative
- Neutral
- Positive
- Strongly positive

Based ONLY on the search results and images presented above, select the level to which you agree or disagree with the following statements.

1. Someone with Down Syndrome is likely to be capable.
2. I find these images/search results inspirational.
3. I find these images/search results upsetting.

*Strongly agree, agree, neutral, disagree, strongly disagree

Based ONLY on the images presented above, select the level to which you agree or disagree with the following statements.

1. Someone with Down Syndrome likely has an easy time making friends.
2. I could envision someone with Down Syndrome engaging in multiple activities within my community.
3. It is possible for someone with Down Syndrome to have a high quality of life.

*Strongly agree, agree, neutral, disagree, strongly disagree

Please carefully review the news headlines below related to Prader-Willi Syndrome.

Please review the following search results related to Prader-Willi Syndrome.

Please review the following images related to Prader-Willi Syndrome.

Based ONLY on the search results and images presented above, my impression of Prader-Willi Syndrome is:

- Strongly negative
- Negative
- Neutral
- Positive
- Strongly positive

Based ONLY on the search results and images presented above, select the level to which you agree or disagree with the following statements.

1. Someone with Prader-Willi Syndrome is likely to be capable.
2. I find these images/search results inspirational.
3. I find these images/search results upsetting.

*Strongly agree, agree, neutral, disagree, strongly disagree

Based ONLY on the images presented above, select the level to which you agree or disagree with the following statements.

1. Someone with Prader-Willi Syndrome likely has an easy time making friends.
2. I could envision someone with Prader-Willi Syndrome engaging in multiple activities within my community.
3. It is possible for someone with Prader-Willi Syndrome to have a high quality of life.

*Strongly agree, agree, neutral, disagree, strongly disagree

Please carefully review the news headlines below related to Angelman Syndrome.

Please review the following search results related to Angelman Syndrome.

Please review the following images related to Angelman Syndrome.

Based ONLY on the search results and images presented above, my impression of Angelman Syndrome is:

- Strongly negative
- Negative
- Neutral
- Positive
- Strongly positive

Based ONLY on the search results and images presented above, select the level to which you agree or disagree with the following statements.

1. Someone with Angelman Syndrome is likely to be capable.
2. I find these images/search results inspirational.
3. I find these images/search results upsetting.

*Strongly agree, agree, neutral, disagree, strongly disagree

Based ONLY on the images presented above, select the level to which you agree or disagree with the following statements.

1. Someone with Angelman Syndrome likely has an easy time making friends.
2. I could envision someone with Angelman Syndrome engaging in multiple activities within my community.
3. It is possible for someone with Angelman Syndrome to have a high quality of life.

*Strongly agree, agree, neutral, disagree, strongly disagree

Do you know someone with Intellectual or Developmental Disability?

- Yes
- No
- Unsure

Do you know someone with (please select all that apply)

- Autism
- Down Syndrome
- Prader-Willi Syndrome
- Angelman Syndrome
- Cerebral Palsy
- None of these

What is your level of education?

- Less than high school
- High school graduate
- Some college
- 4 year degree
- Graduate degree

Do you have children?

- No
- Yes
