# Supplementary material for: Perception of four intellectual and developmental disabilities based on search engine and news portrayal
Source: PLoS One. 2025 Feb 10;20(2):e0316928. doi: 10.1371/journal.pone.0316928 (PMC11809880; doi:10.1371/journal.pone.0316928)
Supplement: Attachment — Submitted filename: Response to Reviewers.docx [file pone.0316928.s011.docx]

Dear PLOS ONE Editors:

We thank the reviewer for their thorough and constructive feedback. We have addressed their concerns and believe we have a stronger and more impactful paper. The summary is below.

1. On page 11 the authors do nor explain why they have chosen the word "inspirational"? It is not clear to me why this term was chosen.

Thank you for this observation. We have addressed this point at the top of page 6 in the manuscript. In short, we chose to ask about perception of images as inspirational due to the common perception of disabled people as “inspirational,” even when doing simple, mundane tasks. This perception, while seemingly positive on the surface to a non-disabled person, feeds into the placing of the burden of access on the individual, as in the medical model of disability (20), by implying a physical or symbolic deficit that must be overcome (16).

2. On page 12 how did the authors arrive at the question where only one question is measuring a negative reaction (upsetting) while the other questions are positive (e.g. doing activities, being capable etc.

We wanted to maximize impact of each question and minimize potential response time. Therefore, we opted to focus more on the positive words and use fewer negative words in our framing. We wanted to use a possible antithesis of the “inspirational” concept to understand if the presence of images as inspirational would yield a perception of images as “not upsetting,” or if these perceptions could be found in tandem.

3. On page 17 the authors state that "an overall more negative portrayal and perception of Prader-Willi
syndrome, compared to Cerebral Palsy, Down syndrome, and Angelman syndrome" On page 18 however they say that " search engine results from Angelman syndrome are perceived more negatively than Down syndrome". this seems contradictory. Perhaps the authors should avoid arbitrary classifications such as "common" and "uncommon" which appear a little arbitrary given that only 4 syndromes featured in this study making such generalisations less credible.

Thank you for this point. Conditions like Prader-Willi syndrome and Angelman syndrome are classified as “rare diseases” by organizations like NIH whereas Down syndrome and CP are not. Nevertheless, our language is imprecise. We have changed common and uncommon to more and less prevalent.

4. On page 19 the authors state "Findings show that increased awareness of the social model and disability perspectives can improve physician care for patients and colleagues with disabilities". This is an important statement and perhaps needs more emphasis.

We have added this to the abstract to stress the importance of this finding.

Thank you again for your insightful comments. With these modifications, we believe our manuscript will contribute even more impactfully to the PLOS ONE community and appreciate the opportunity to resubmit.

Best Regards,

Lillian Droscha, MPH
